# *De Novo* Assembly and Discovery of Genes That Are Involved in Drought Tolerance in Tibetan *Sophora moorcroftiana*

**DOI:** 10.1371/journal.pone.0111054

**Published:** 2015-01-05

**Authors:** Huie Li, Weijie Yao, Yaru Fu, Shaoke Li, Qiqiang Guo

**Affiliations:** 1 Agricultural and Animal Husbandry College, Tibet University, Nyingchi, Tibet, China; 2 Key Laboratory of Forest Ecology in Tibet Plateau (Tibet University), Ministry of Education, Nyingchi, Tibet, China; 3 National Key Station for Field Scientific Observation & Experiment, Nyingchi, Tibet, China; Institute of Crop Sciences, China

## Abstract

*Sophora moorcroftiana*, a Leguminosae shrub species that is restricted to the arid and semi-arid regions of the Qinghai-Tibet Plateau, is an ecologically important foundation species and exhibits substantial drought tolerance in the Plateau. There are no functional genomics resources in public databases for understanding the molecular mechanism underlying the drought tolerance of *S. moorcroftiana*. Therefore, we performed a large-scale transcriptome sequencing of this species under drought stress using the Illumina sequencing technology. A total of 62,348,602 clean reads were obtained. The assembly of the clean reads resulted in 146,943 transcripts, including 66,026 unigenes. In the assembled sequences, 1534 transcription factors were identified and classified into 23 different common families, and 9040 SSR loci, from di- to hexa-nucleotides, whose repeat number is greater than five, were presented. In addition, we performed a gene expression profiling analysis upon dehydration treatment. The results indicated significant differences in the gene expression profiles among the control, mild stress and severe stress. In total, 4687, 5648 and 5735 genes were identified from the comparison of mild versus control, severe versus control and severe versus mild stress, respectively. Based on the differentially expressed genes, a Gene Ontology annotation analysis indicated many dehydration-relevant categories, including ‘response to water ‘stimulus’ and ‘response to water deprivation’. Meanwhile, the Kyoto Encyclopedia of Genes and Genomes pathway analysis uncovered some important pathways, such as ‘metabolic pathways’ and ‘plant hormone signal transduction’. In addition, the expression patterns of 25 putative genes that are involved in drought tolerance resulting from quantitative real-time PCR were consistent with their transcript abundance changes as identified by RNA-seq. The globally sequenced genes covered a considerable proportion of the *S. moorcroftiana* transcriptome, and the expression results may be useful to further extend the knowledge on the drought tolerance of this plant species that survives under Plateau conditions.

## Introduction

The Qinghai-Tibet Plateau is generally called “the roof of the world” because of its extremely high altitude and extreme environment. The Plateau plays an important role in determining the formation and variation of regional weather and climate in East and South Asia, as well as the Northern Hemisphere atmospheric circulation in general [1]. It was recently discovered that the dry and warming climate and other factors have caused desertification expansion in some portions of the Plateau [2], [3].

*Sophora moorcroftiana* is an endemic Leguminosae shrub species that is restricted to the arid and semi-arid regions of the Plateau. *S. moorcroftiana* mainly grows in the middle and upper reaches of the dry valley region of the Yarlung Tsangpo River at high altitudes ranging from 2,800 m to 4,400 m. This species is a unique *Sophora* characterized in this area by strong drought resistance after long-term adaptation to the local environment and climate. This species is currently the preferred drought-resistant afforestation tree species in the sand land in the Plateau.

With the development of molecular technologies and ‘omics’ tools, studies of the transcriptome and Differential Expression Genes (DEGs) under certain condition have become powerful strategies for the global analysis of plant genes. Using transcriptome and DEG data, the abiotic stress response of *Arabidopsis* and other non-model plants has been widely studied [4]–[9]. However, until now, no genes have been identified, and no molecular research of this species has been reported, despite the importance of the genus. Considering the large genome size of the plant, the whole genome sequencing of *S. moorcroftiana* is difficult; therefore, the construction of large EST collections of this species is the most promising approach for providing functional-genomics-level information in *S. moorcroftiana*.

In this study, in order to identify drought-tolerant genes, potential dehydration-responsive genes were first identified based on Illumina tag-sequencing, and then, the DEGs were screened and further validated by qRT-PCR. This study will be helpful for elucidating the molecular responsive mechanism of *S. moorcroftiana* to drought stress and for further improving drought resistance by genetic modification in the future.

## Results and Discussion

### Leaf water potential during drought stress

To evaluate the drought stress conditions, three trees were selected for each treatment, and three biological replicates were set. Water stress was imposed by withholding water until a desired stress condition was reached. The standard used for differentiating the mild drought- stressed and severe drought- stressed trees was the leaf water potential. The potential of control trees was between -0.9 and -1.1 MPa, (2) while the mild drought-stressed wasbetween-1.9 and -2.1 MPa, and (3) trees the severe drought-stressed was between 3.0 and 3.2 MPa. The lower leaves of the severely stressed trees were slightly wilted, while the leaves of the mildly stressed and the control trees experienced normal growth.

### Transcriptome sequencing and assembly

**Figure 1:**
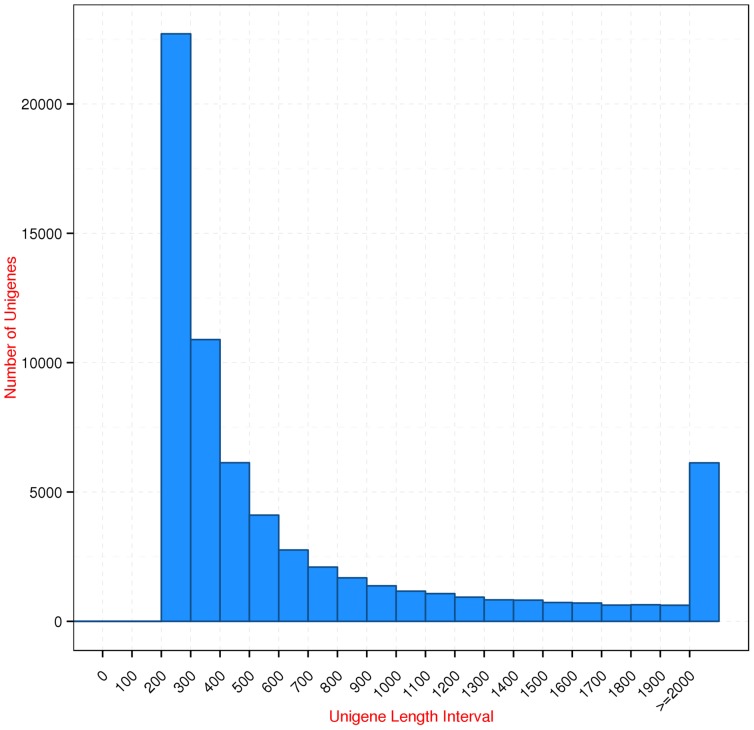
Length distribution of the assembled unigenes.

Transcriptome sequences are valuable resources, especially for species without a sequenced genome, such as the non-model plant species *S. moorcroftiana*. These sequences accelerate gene discovery, permitting expression analysis evolutionary genome dynamics studies. In this study, Next-Generation Sequencing enabled the generation of large numbers of sequence reads in a rapid and cost-effective manner. The Illumina sequencing data of *S. moorcroftiana* were deposited into the NCBI SRA database under the accession number SRP041237. A total of 63,855,218 raw reads were generated. After removing the low-quality reads and trimming off the adapter sequences, 62,348,602 clean reads were obtained. The assembly of the clean reads resulted in 146,943 transcripts, including 66,026 unigenes that ranged from 201 to 17064 bp with an N50 length of 1438 bp (Table 1). The length statistics of the assembled unigenes were displayed (Figure 1). To our knowledge, this is the first report of *S. moorcroftiana* transcriptome data.

**Table 1:** Summary of sequences analysis.

Description	Number
Before trimming	
Raw reads	63,855,218
After trimming	
Clean reads	62,348,602
Clean bases (Gb)	6.24
GC ontent (%)	40
Q20 percentage (%)	98.53
After assembly	
Unigenes	66,026
Min length (bp)	201
Max length (bp)	17,064
Average length (bp)	773
N50 (bp)	1438
N90 (bp)	291

### Blast analysis

To predict and analyze the function of the assembled transcripts, non-redundant sequences were submitted to a BLASTx search against the following databases: Nr (NCBI non-redundant protein sequences), Nt (NCBI non-redundant nucleotide sequences), Pfam (Protein family), Swiss-Prot (a manually annotated and reviewed protein sequence database), GO (Gene Ontology), KOG (eukaryotic orthologous groups) and KEGG (Kyoto Encyclopedia of Genes and Genomes). The unigenes were subjected to public databases for similarity searching. Among these unigenes, 31,117 (47.12%), 20,646 (31.26%) and 21,493 (32.55%) unigenes showed identity with sequences in the NCBI Nr, Nt and SwissProt databases, respectively, with an e value <1e-5.

### Functional annotation and pathway assignment

**Figure 2:**
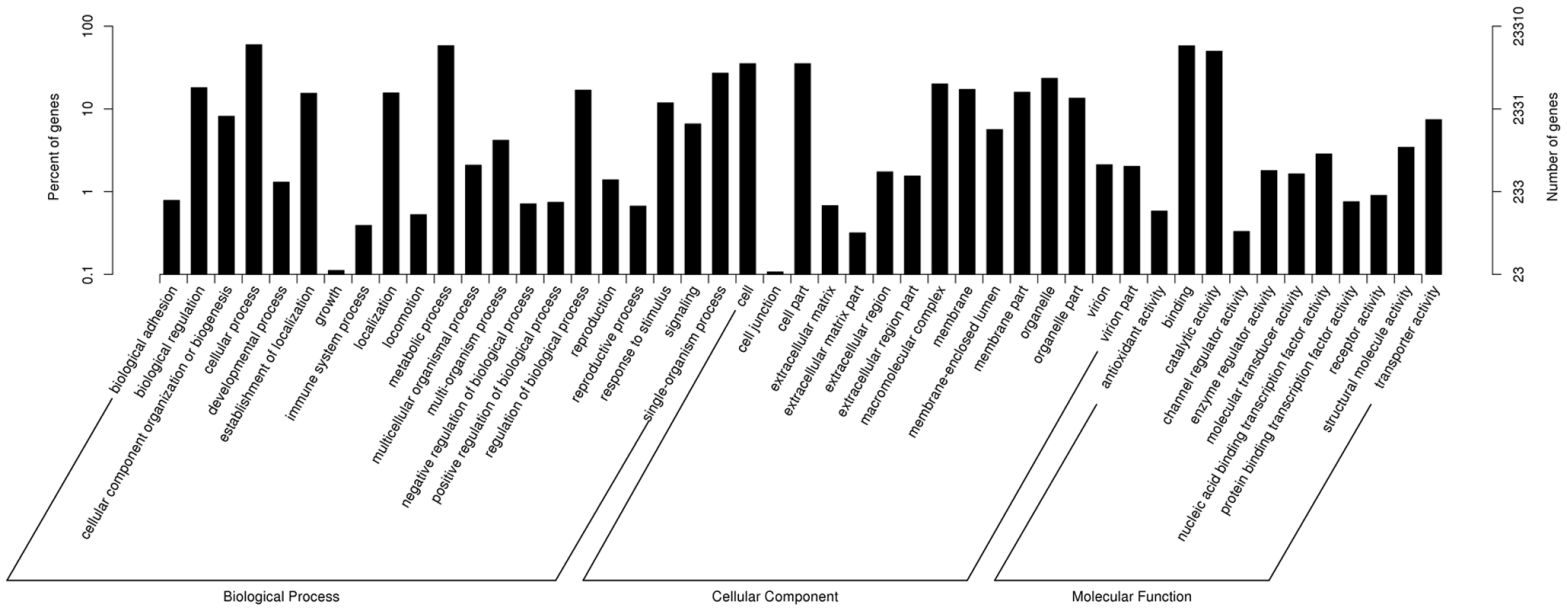
Gene categorization of the assembled unigenes. The unigenes with the best BLAST hits were aligned. All 23,310 unigenes were classified into three major functional categories and 47 sub-categories. The right Y-axis represents the number of genes in a category; the left Y-axis indicates the percentage of a specific category of genes in each main category.

**Figure 3:**
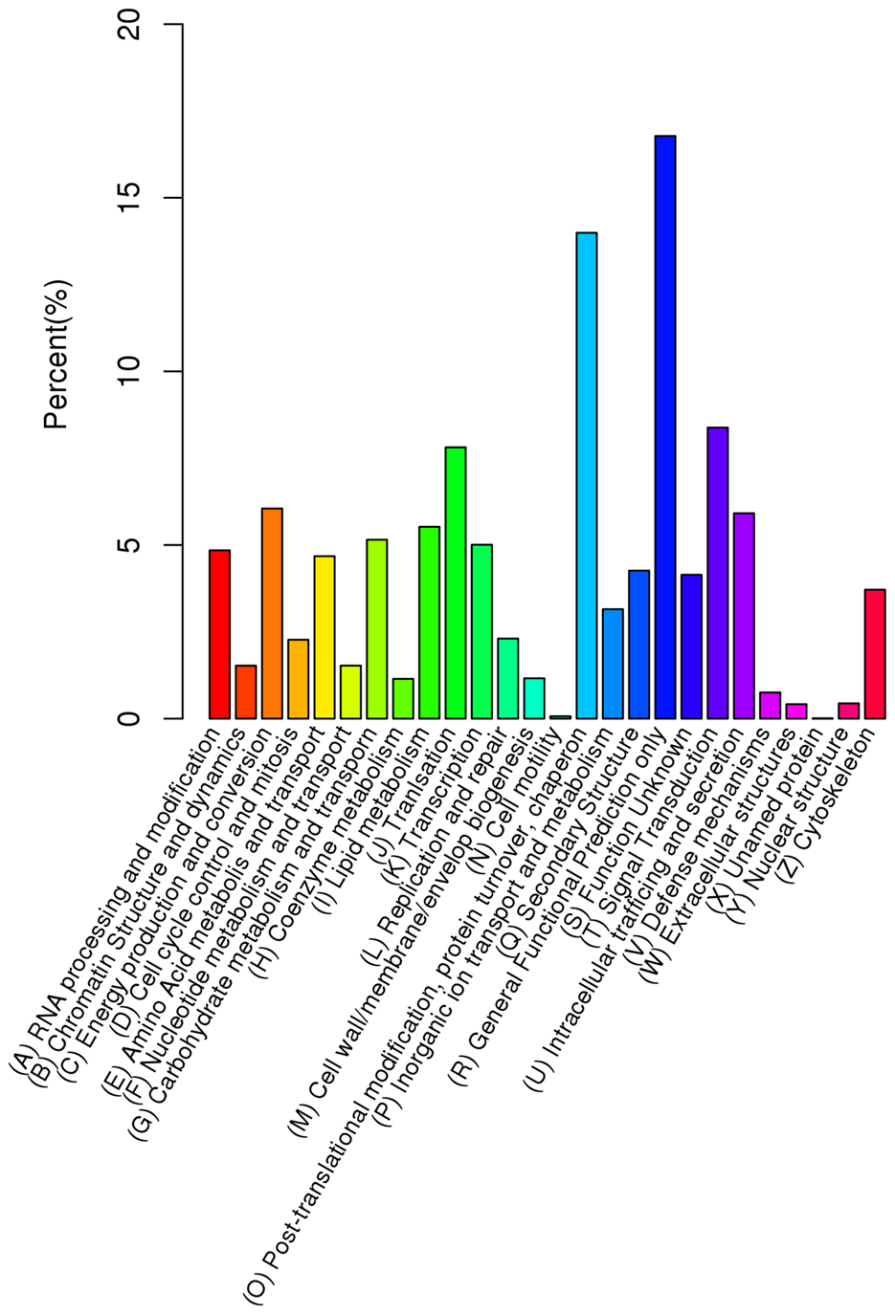
KOG classification of the putative proteins. All 11,801 unigenes were subdivided into 26 eukaryotic Orthologous Group (KOG) classifications. The Y-axis indicates the number of unigenes in a specific functional cluster.

**Figure 4:**
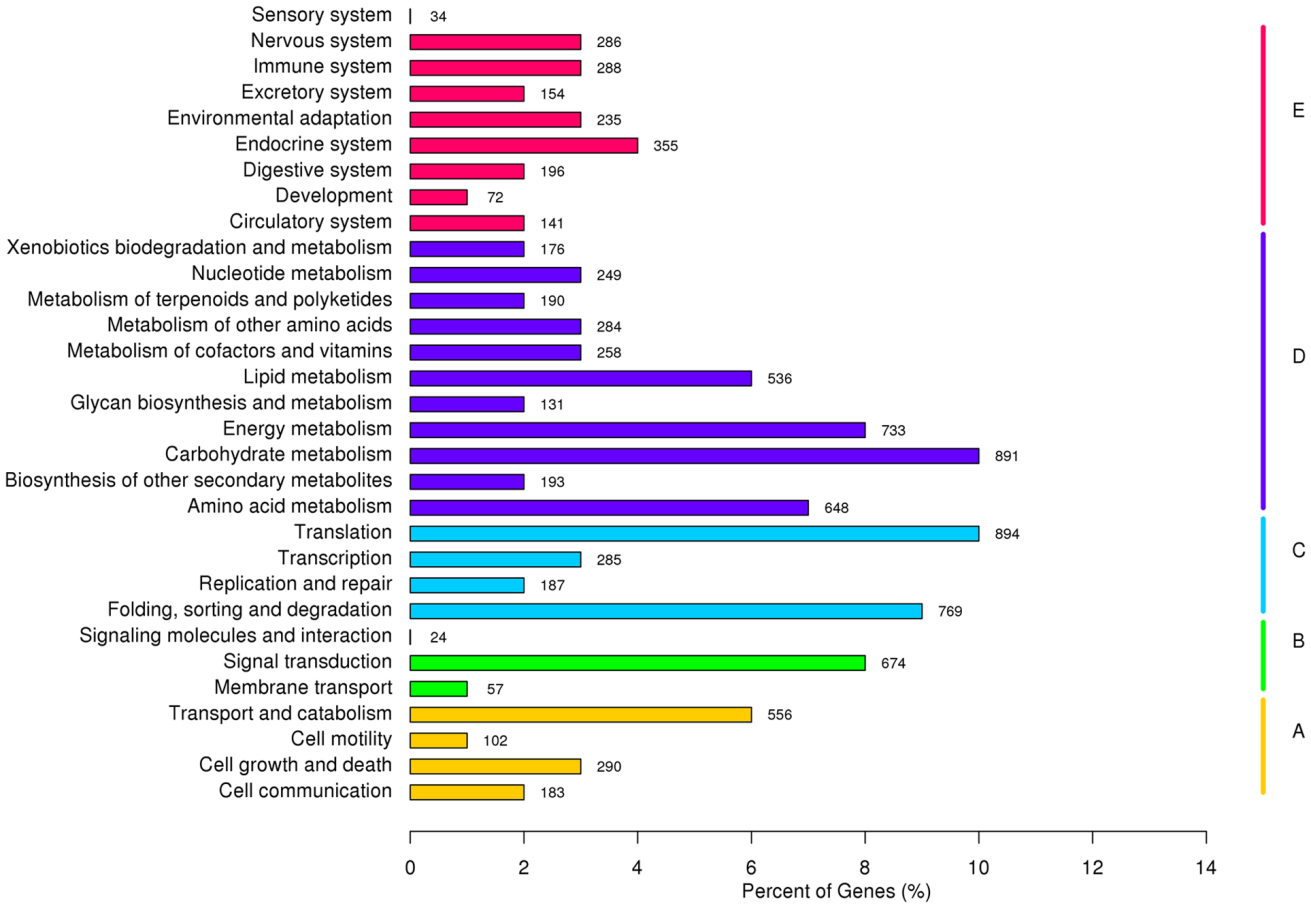
Histogram presentation of the KEGG classification of the annotated transcripts. The left Y-axis indicates the KEGG pathway. The right Y-axis indicates the sub-branches. A, cellular processes; B, environmental information processing; C, genetic information processing; D, metabolism; E, organismal systems. The X-axis indicates the percentage of unigenes that were assigned to a specific pathway.

Gene Ontology (GO) assignments were used to classify the functions of the predicted *S. moorcroftiana* genes; 23,310 (35.3%) unigenes were classified into three major functional categories (Biological Process, Cellular Component and Molecular Function) and 47 subcategories (Figure 2). In terms of Biological Processes, ‘metabolic processes’ and ‘cellular processes’ were the top two GO terms, indicating that the leaves were undergoing extensive metabolic activities, which is consistent with the results from the leaves of another Leguminosae genus *Prosopis alba* in Argentina [10]. In terms of Molecular Function, the top three GO terms were related to the following categories: ‘binding’, ‘catalytic activity’ and ‘transporters activity’. A detailed analysis of the Cellular Component showed that the most representative categories were ‘cell part’, ‘organelle’ and ‘macromolecular region part’. These results are similar with the GO assignments of transcriptome data from abiotic-tolerant *Reaumuria trigyna*, *Prosopis alba*, *Anthurium*, and *Chorispora bungeana* [9]–[12]. To classify the orthologous gene products, 11,801 (17.87%) unigenes were subdivided into 26 eukaryotic Orthologous Groups (KOG) classifications (Figure 3). Among these classifications, the cluster of ‘general function prediction only’ represented the largest group, followed by ‘post-translational modification’, ‘protein turnover’, ‘chaperon’, ‘translation’ and ‘signal transduction’. The two categories involving ‘cell motility’ and ‘unnamed protein’ represented the smallest KOG classifications (Figure 3). To identify the biological pathways in the annotated sequences using the Kyoto Encyclopedia of Genes and Genomes (KEGG), the assembled unigenes were assigned to five specific pathways, including Cellular Processes, Environmental Information Processing, Genetic Information Processing, Metabolism, and Organism Systems (Figure 4). A summary of the unigenes annotation is given in Table S1.

### Transcription factors

**Figure 5:**
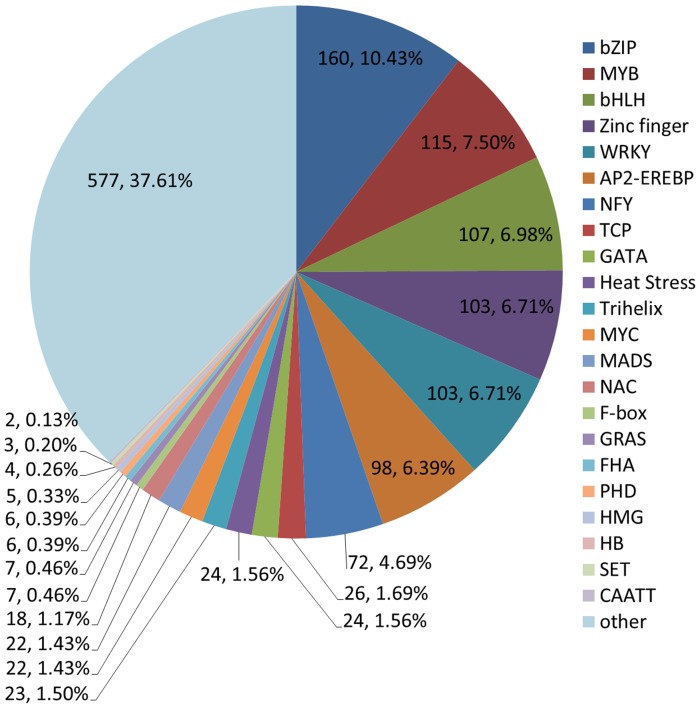
Number of unique transcripts that were annotated as transcription factors.

Transcription factors (TFs) are important upstream regulatory proteins and play significant roles in plant responses to abiotic and biotic stresses. In this study, 1534 TFs were identified and classified into 23 different common families by searching from unique transcripts (Figure 5). The largest group of TFs was the bZIP family (160, 10.43%), followed by MYB (115, 7.5%), bHLH (107, 6.98%), zinc finger (103, 6.71%), and WRKY (103, 6.71%). This result is similar to the results from the *Arachis hypogaea* transcriptome, whose largest group is bZIP, followed by MYB, NAC, bHLH, AP2-EREB and WRKY [13], and similar to the results from the *Chrysanthemum morifolium* transcriptome, whose largest group is MYB, followed by C3H, AP2, C2H2, bHLH and the WRKY big groups [14]. These results further suggest that bZIP, MYB, bLHL and WRKY TFs are superfamilies in plants, and expression of most numbers are affected by abiotic stress [4], [8], [14].

### SSR identification

SSRs have much higher levels of polymorphisms than do most other marker systems due to their codominance, hypervariability, high reproducibility and abundance in eukaryotic genomes. The utility of SSRs in genetic studies is well established [15], [16]. In this study, the EST-SSRs in the transcriptome of *S. moorcroftiana* were discovered based on an analysis of the assembled contig templates. A total of 12,086 distinct SSR loci were identified. Among these loci, SSR loci from di- to hexa-nucleotide, whose repeat number is greater than five, accounted for 9040. Most of these satellites are di- or tri-nucleotide motifs, being 2182 and 2199, respectively (Table 2). The AG/CT was the most frequent di-nucleotide SSR repeat and accounted for 1326, and the AAG/CTT was the most frequent tri-nucleotide SSR repeat and accounted for 568. Details of the identified SSRs are listed in Table S2. Likewise, AG/CT and AAG/CTT were the most frequent di- and tri-nucleotide SSR repeats in *Ammopiptanthus mongolicus* [17], which might be due to the fact that both *S. moorcroftiana* and *A. mongolicus* belong to legume plants and may contain similar SSR characteristics in their genomes.

**Table 2:** Summary of EST-SSR detected from transcriptome data.

Motif length	Repeat numbers							Total(%)	
	5	6	7	8	9	10	>10		
Di-	762	453	354	267	246	92	8	2182	48.27%
Tri-	1335	565	264	33	-	-	2	2199	48.65%
Tetra-	96	19	-	-	-	2	1	118	0.38%
Penta-	17	-	-	-	-	-	-	17	0.38%
Hexa-	-	1	1	-	1	-	1	4	0.09%
Total	2210	1038	619	300	247	94	12	4520	

### Comparison of the unigenes that are differentially expressed under drought stress

**Figure 6:**
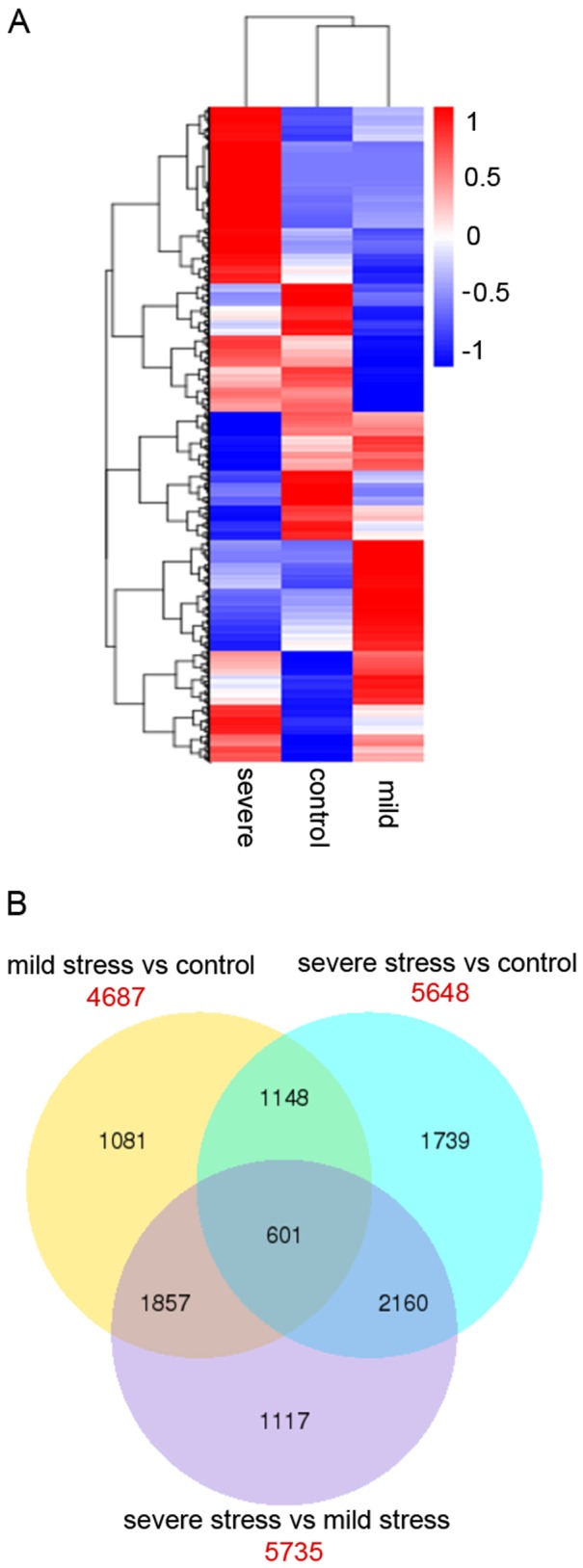
Cluster analysis of the DEGs across three comparisons. A. The differentially expression levels were log10 transformed and are shown with high expression represented by red and low expression represented by blue. B. Venn diagrams showing DEGs across three comparisons (mild stress versus control; severe stress versus control; and severe stress versus mild stress). The red values correspond to the total number of DEGs in each comparison; the overlapping values correspond to the number of differentially expressed genes in two/three comparisons.

The expression of a high number of unigenes was affected in the drought-treated trees. Only the genes whose expression was identified as being significantly changed with a p value-adjusted (pdaj) <0.05 were retained. To analyze the similarities and differences among the drought-responsive transcriptome, a hierarchical clustering was prepared to represent the transcripts of all of the DEGs in the three replicates of the control, mild stress and severe stress trees. These results indicated significant differences in the gene expression profiles among the control, mild stress and severe stress (Fig 6A). When the DEGs were compared under the three conditions, we discovered 4687, 5648 and 5735 genes in the comparisons of mild versus control, severe versus control and severe versus mild stress, respectively. Notably, more genes were differentially expressed under severe stress compared to mild stress, suggesting that a severe stress treatment could affect more drought stress-related genes than mild stress. Moreover, a total of 601 genes overlapped with those of mild versus control, severe versus control and severe versus mild stress, indicating a linkage among three comparisons and a progressive biological process (Fig. 6B).

### Functional classification of the drought-stressed genes by Gene Ontology analysis

**Figure 7:**
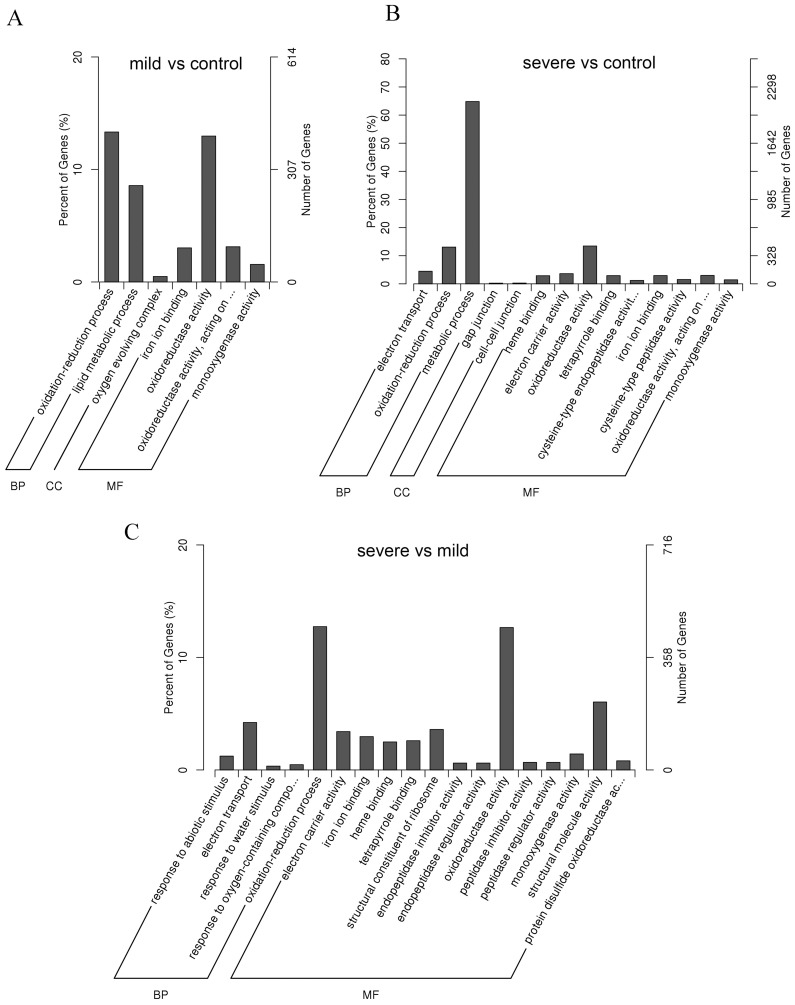
GO classifications of DEGs across three comparisons. The right Y-axis represents the number of DEGs in a category; the left Y-axis indicates the percentage of a specific category of DEGs in each main category. A, mild stress versus control; B, severe stress versus control; C, severe stress versus mild stress.

To identify the genes that are differentially expressed under drought stress, a functional categorization was carried out by GO analysis. By comparing mild stress versus control, 3068 DEGs, including 1223 down-regulated genes and 1845 up-regulated genes, revealed by DEG analysis were functionally assigned to the relevant terms in three categories (Biological Process, Cellular Component, and Molecular Function) of the GO database. The GO terms of the ‘oxidation-reduction process’ in Biological Process and ‘oxidoreductase activity’ in Molecular Function were significantly overrepresented (Figure 7A). By comparing severe stress with control, 3283 DEGs, including 1200 down-regulated genes and 2083 up-regulated genes, were functionally assigned to the relevant terms; ‘metabolic process’ in Biological Process was significantly overrepresented, followed by ‘oxidation-reduction process’ in Biological Process and ‘oxidoreductase activity’ in Molecular Function (Figure 7B). By comparing severe stress with mild stress, 3579 DEGs, including 1811 down-regulated genes and 1768 up-regulated genes, were functionally assigned to the relevant terms. Similar to the above two comparisons, the ‘oxidation-reduction process’ in Biological Process and ‘oxidoreductase activity’ in Molecular Function were significantly overrepresented (Figure 7C). These data, overall, suggest that ‘oxidation-reduction’ and ‘oxidoreductase activity’ were strongly affected may due to that the drought stress-induced generation of active oxygen at the cellular level is tightly controlled at both the production and consumption levels *in vivo* through increased antioxidative systems to avoid drought injury under drought conditions [18].

In addition, as expected, the GO terms ‘response to water stimulus’, ‘response to water deprivation’, ‘hyperosmotic response’, and ‘response to stress’ were highly enriched in the DEGs, further confirming the efficiency of the drought treatments and the reliability of the gene expression data. At the same time, other terms that were related to the response to various other types of abiotic and biotic stresses, such as ‘response to abiotic stimulus’ ‘hyperosmotic salinity response’, ‘response to temperature stimulus’, and ‘immune response’, were also highly enriched in the DEGs, indicating the crosstalk of different stress responses in *S. moorcroftiana*, similar to the previously reported plant species [9], [14]. Furthermore, a general induction and enhancing of gene expression occurred under drought stress. Moreover, the DEGs were classified into additional GO terms of Molecular Function, which might due to more genes being differentially expressed in severe stress compared to mild stress (Figure 6B). However, for the Cellular Component category, dozens of DEGs were identified by comparing mild stress with control, while several DEGs were identified by comparing severe stress with control, and no DEG was identified by comparing severe stress with mild stress, suggesting that the expression of the unigenes that are involved in Cellular Component were not obviously changed under drought stress despite the degree of drought stress.

### KEGG pathway analysis of the drought-responsive genes

**Figure 8:**
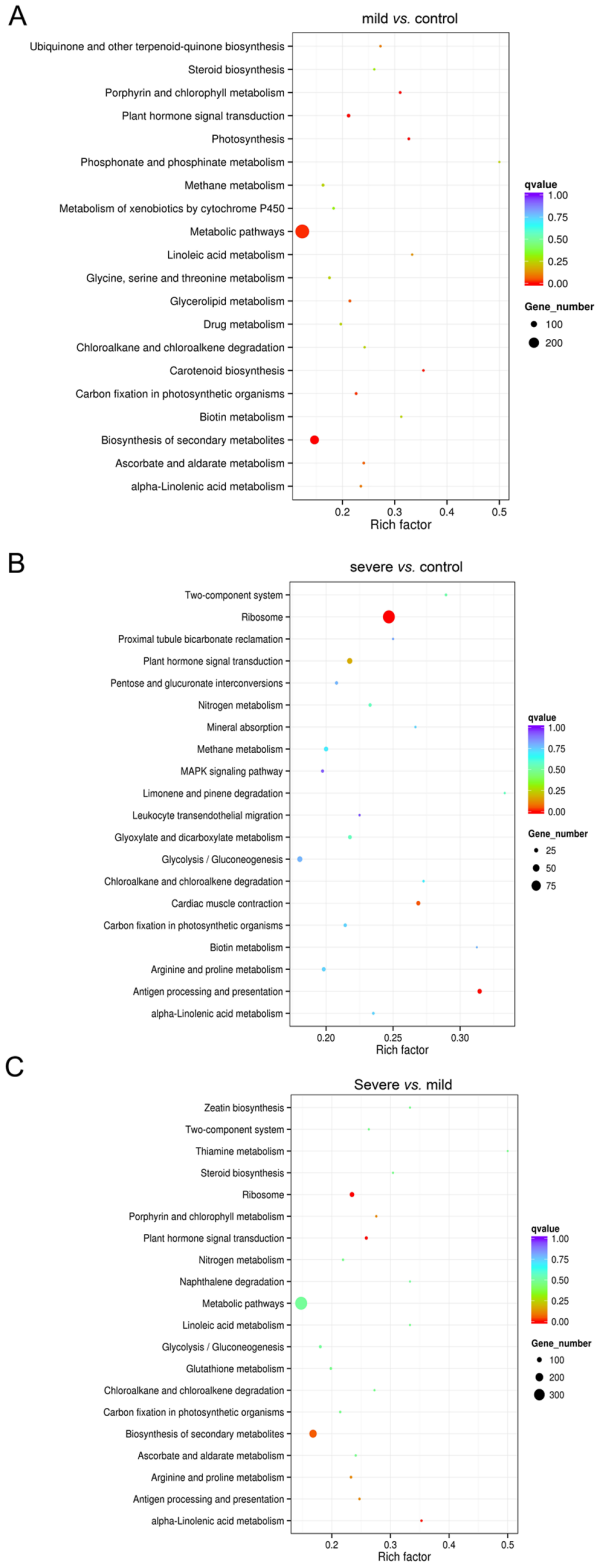
KEGG enrichments of the annotated DEGs across three comparisons. The left Y-axis indicates the KEGG pathway. The X-axis indicates the Rich factor. A high q value is represented by blue, and a low q value is represented by red. A, mild stress versus control; B, severe stress versus control; C, severe stress versus mild stress.

To determine whether the drought stress-responsive genes engaged in specific pathways, the DEGs were used as objects to search against the KEGG pathway database. The top 20 obviously enriched pathways are shown in Fig. 8. By comparing mild stress with control, the DEGs were enriched in ‘metabolic pathways’, and 165 DEGs were related to ‘biosynthesis of secondary metabolites’ (Fig. 8A). However, this is only approximately 12% and 15% of the total genes that are involved in ‘metabolic pathways’ and ‘biosynthesis of secondary metabolites’ (Table S3), respectively. Although, only 52 and 58 genes that are related to the pathways ‘photosynthesis’ and ‘porphyrin and chlorophyll metabolism’, more than 30% DEGs were enriched (Table S3), suggesting that mild drought affected plant photosynthesis, which also occurs in other plant species under drought, including *C. morifolium* [14], *B. nivea* [4], and *A. mongolicus* [17]. By comparing severe stress with control, among the genes that are related to the pathways ‘ribosome’ and ‘plant hormone signal transduction’ (Fig. 8B), approximately 25% and 22% of the DEGs were enriched, respectively (Table S4). By comparing severe stress with mild stress, similar to the results of comparing mild stress with control, more DEGs were enriched in the pathways ‘metabolic pathways’ and ‘biosynthesis of secondary metabolites’ (Fig. 8C). However, the enrichment was only approximately 15% and 17%, respectively. In addition, among the genes that are related to the pathways ‘ribosome’, ‘plant hormone signal transduction’, ‘ascorbate and aldarate metabolism’, and ‘carbon fixation in photosynthetic organisms’, approximately 24%, 26%, 24%, and 22% of the DEGs were enriched, respectively (Table S5). These results are consistent with drought-stressed *B. nivea*, in which the ‘ribosome’ pathway enriched the most DEGs, followed by ‘ascorbate and aldarate metabolism’ and ‘carbon fixation in photosynthetic organisms’ and other pathways [4]. In general, these data indicate that severe stress strongly affects ‘ribosome’, ‘plant hormone signal transduction’, ‘ascorbate and aldarate metabolism’, and ‘carbon fixation in photosynthetic organisms’ in *S. moorcroftiana*, possibly due to the drought stress decreasing the CO_2_ assimilation rates that because of reduction of stomatal conductance, decreases the contents and activities of photosynthetic carbon reduction cycle enzymes, and induces plant hormone signal transduction [18], [19]. In addition, severe drought may affect the transcription of genes by changing the expression of ‘ribosome’-related genes.

### Expression analysis of the genes that are potentially involved in the drought response

To identify drought-responsive genes, 25 unigenes were selected. The expression of 25 unigenes was significantly up-regulated or down-regulated under drought treatment.

**Figure 9:**
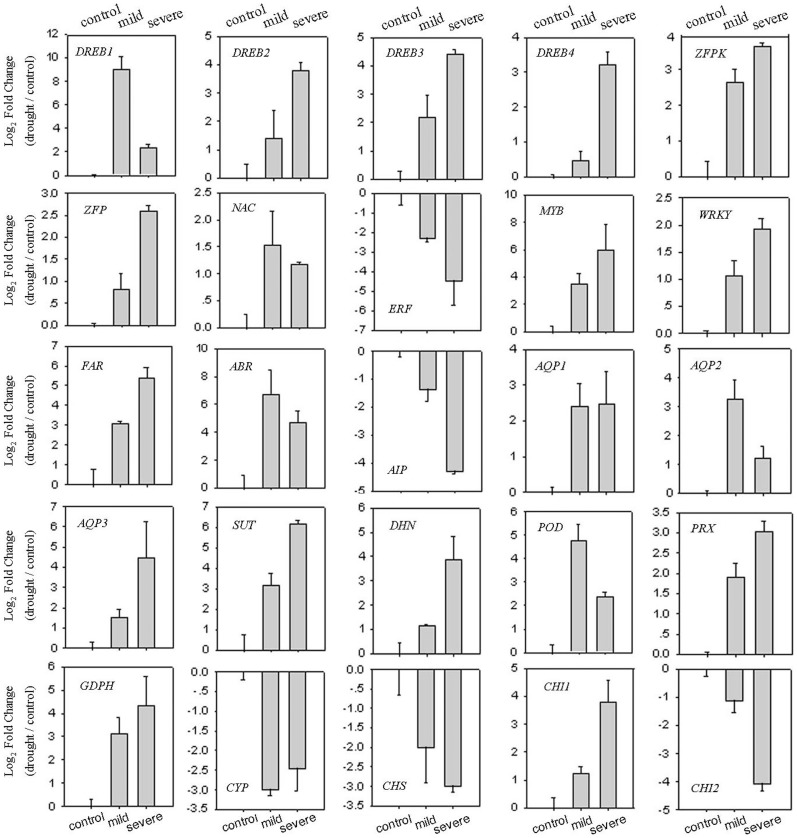
Verification of 25 putative genes that are involved in the drought response by qRT-PCR. The left Y-axis indicates the normalized expression pattern of the DEGs under drought. The normalized expression patterns were log2 transformed. Both *Actin* and *GAPDH* were used as internal controls.

*Transcription factors*. Transcription factors are widely involved in drought stress, especially DREB TFs. In this study, 1534 TFs were identified from the transcriptome data, many of which were differentially expressed among the control and drought-stressed leaves. Eleven TFs were selected for further expression analysis; all seven were drought-responsive TFs (Fig. 9). Among these TFs, nine genes encoding DREB, Zinc-Finger Protein (ZFP), Zinc-Finger Protein Kinase (ZFPK), MYB, NAC, and WRKY were induced, while an *ERF* was repressed by drought; these results indicate that TFs are widely involved in plant response to drought stress, and most of these TFs fall into the AP2/ERF, NAC, MYB, Zinc-Finger and WRKY superfamilies [20], [21]. Consistently, 12 TFs from *B. nivea*, including AP2, NAC, MYB, Zinc-Finger, and WRKY, were drought stress-responsive TFs [4]. Eight *DREB* genes were responsive to dehydration in chrysanthemum [14], and two genes encoding *NAC* and *ERF* were up-regulated in *A. mongolicus* [17]. In addition, expression of AP2/ERF, NAC, MYB, Zinc-Finger, WRKY and other TF genes were induced in drought-tolerant rice under drought [22]. It is worth noting that a recent study reported a novel TF, named Far-Red impaired response 1 (FAR1), which is derived from ancient mutator-like transposases and belongs to the *FRS* gene family in *Arabidopsis*, that was remarkably up-regulated by drought treatment [23]; our results also demonstrate that the *FAR* gene was induced by drought in *S. moorcroftiana*, suggesting that *FAR1* may be involved in additional processes in plants.

#### Regulating plant hormones

Extensive overlaps exist between the drought response and several plant hormone responses, including auxin and ABA in *Arabidopsis* [24]. The promoters of many drought-inducible genes contain the ABA-responsive element, including some TFs; 67% of drought-regulated genes are significantly regulated by ABA [25]. In this study, the expression of an ABA-Responsive (ABR) gene was induced by drought, suggesting that the drought response may be linked to ABA signaling. In contrast, the expression of a gene encoding Auxin-Induced Protein (AIP) in *S. moorcroftiana* was down-regulated under drought; our data agree with the observation that auxin-responsive genes were down-regulated by drought in *Arabidopsis* [25], suggesting that *S. moorcroftiana* drought tolerance also linked to the auxin signaling network.

#### Adjust the osmotic pressure

Water-channel proteins and sugar transporters are believed to function in the transport of water and sugars to adjust the osmotic pressure under stress [26]. The transcripts of most *Aquaporins* (*AQPs*) were elevated under drought stress in *Malus* species [27]. In addition, at least five *AQPs* were up-regulated in rice under drought [28]. These studies suggest that the expression of *AQPs* may be induced by drought; however, the transcripts some *AQPs* were down-regulated in rice, sugarcane, and Chinese cabbage under drought [28]–[30]. In this study, three selected *AQPs* were induced by drought (Fig. 9). These results indicating that the role of *AQPs* in drought stress is complex. Moreover, the transcript of a gene encoding Sugar Transporter (SUT) was induced by drought in this study (Fig. 9), which could be explained by additional sugar transportation adjusting the osmotic pressure under drought.

#### Stabilizing cell structures

Dehydrins (DHNs) are a class of hydrophilic, thermostable stress proteins with a high number of charged amino acids. DHNs function to protect cells from damage caused by stress-induced dehydration [31]. The expression of *DHN*s is usually induced by drought stress. For example, the transcripts of *DHNs* were strongly up-regulated by drought stress in peach and tobacco [32], [33]. Similarly, similar to previous reports, a selected *DHN* from *S. moorcroftiana* was also induced by drought in this study. However, this is not always the case, as some *DHNs* also remained unchanged under drought in grape and barley [34]–[36], possibly resulting from differences in the *DHNs* expression levels often being dependent on the duration of the stress.

#### Reducing the oxidative damage

The induction of Reactive Oxygen Species (ROS)-related factors suggests that drought stress is accompanied by the production of ROS in plant roots, and the induction of ROS-related enzymes may be involved in the protection of tissues from oxidative damage under stress conditions [37]. The response and dynamics of antioxidant enzyme transcripts, including *SOD*, *POD*, *PRX*, *APX* and *GR* et al., are commonly used to study plant stress responses. Of the antioxidative enzymes, Peroxidases (POD) play key roles in cellular ROS detoxification, Peroxiredoxins (PRX) are a recently discovered family of antioxidant enzymes that catalyze the reduction of peroxides. In the woody plant species *Tamarix hispida*, transcripts of 10 *PODs* were highly induced by drought in different organs [38]. In agreement with this observation, a *POD* was obviously induced under drought in this study. Moreover, the expression of a *PRX* of *Xerophyta viscosa* was up-regulated when exposed to dehydration [39]. However, the expression patterns of four *PRXs* showed temporal and organ specificity under PEG treatment in *X. viscosa*; all four *PRXs* were down-regulated in the leaves, and three of them were generally decreased in the roots, while three *PRXs* was up-regulated in the stems under PEG stress [40]. Similar with these results, expression of *PRX* was significantly increased in the leaves under drought. In addition, in *Arabidopsis*, deficiency of Glycerol-3-phosphate dehydrogenase (GPDH) leads to a higher cellular level of ROS, and a noticeable increase in the *GPDH* transcript was observed after exposing the trees to drought stress [41]. The expression pattern was also observed in this study, in which a *GPDH* was increased under drought in *S. moorcroftiana*. These results suggest that the *POD*, *PRX* and *GPDH* in *S. moorcroftiana* are drought-induced genes. *Arabidopsis* contains 244 Cytochrome 450 (CYP450) genes in its genome; transforming *Arabidopsis AtCYP78A7* into rice increased the drought tolerance of the transgenic rice [42], whereas the disruption of *CYP707A3* in *Arabidopsis* results in increased drought tolerance, and its over-expression results in an increased transpiration rate and reduced drought tolerance [43], suggesting that different plant *CYPs* could be positive or negative regulators of drought tolerance. In this study, drought stress increased the transcripts of a selected *CYP* gene, indicating that this *CYP* may be a positive regulator of drought tolerance in *S. moorcroftiana*.

#### Secondary metabolism

The transcripts of some key enzymes that are related to important secondary metabolisms were significantly affected by drought. Chalcone isomerase (CHI) and chalcone synthase (CHS) are two key enzymes in the biosynthesis of flavonoids in plants. In chrysanthemum, the transcripts of *CHS* and *CHI* were down-regulated by drought [14]. However, a rapid increase in the expressions of *CHS* and *CHI* was observed under drought in two wheat cultivars [44]. Interestingly, *CHS* and *CHI* were up-regulated following short-term dehydration stress in roots [45], whereas they were repressed during long-term drought in the roots of alfalfa [46]. In this study, one *CHI* was induced by drought, while another selected *CHI* and a *CHS* were repressed by drought in *S. moorcroftiana*. This discrepancy might reflect differences in the function of plant *CHI* and *CHS* in response to drought stress.

In summary, the expression patterns of 25 putative genes that are involved in drought tolerance were consistent with changes in their transcript abundance, mainly through regulating hormone signaling, reducing oxidative damage, adjusting the osmotic pressure, and regulating secondary metabolism. Meanwhile, transcription factors play important roles in drought tolerance in *S. moorcroftiana*.

## Conclusions

The combination of RNA-seq and DEGs analyses based on Illumina sequencing technology provided comprehensive information on gene expression. The substantially assembled sequences represented a considerable portion of the transcriptome of *S. moorcroftiana*. Based on the assembled *de novo* transcriptome, 4687, 5648 and 5735 DEGs were identified from the comparison of mild versus control, severe versus control and severe versus mild stress, respectively. Kyoto Encyclopedia of Genes and Genomes pathway analysis uncovered the differentially expressed genes were involved in important pathways, such as ‘metabolic pathways’, and ‘plant hormone signal transduction’. In addition, expression patterns of 25 selected DEGs were further validated with qRT-PCR, which reflected significant alteration in major biological processes and metabolic pathways during drought stress. The information provided here can also further extend the knowledge of the drought tolerance of this plant species that survives in the arid and semi-arid regions of the Qinghai-Tibet Plateau.

## Methods

### Plant material and stress treatment

This study was approved by the National Key Station for Field Scientific Observation & Experiment. This field studies did not involve endangered species. The seeds of *S. moorcroftiana* were identified and collected by Yanhui Ye (Agricultural and Animal Husbandry College, Tibet University) in Oct. 2010 from Milin County (N 29^o^12′24.58″, E 94^o^12′2.29″, H 2936 m), Nyingchi, Tibet. To elucidate the drought tolerance mechanism under natural Plateau conditions, two-year-old *S. moorcroftiana* trees (approximately 60 cm in height) in pots were selected from germplasm nursery (field conditions) of the Agricultural and Animal Husbandry College, Tibet University. The trees were randomly assigned to one of three different treatments: (1) control trees maintained under well-watered conditions, (2) mildly drought-stressed trees and (3) severely drought-stressed trees. The water potential was assessed by repeated measurements of the predawn leaf water potential using the PSYPRO Water potential system with C52 psychrometer (Wescor, USA) following the manufacturer's protocol.

### Total RNA isolation and RNA-seq library construction

For the samples for RNA-seq, the leaves from the middle position of the trees were collected from the control and stressed plants and then immediately placed in liquid nitrogen. The total RNA was isolated using EASYspin Plus Plant RNA isolation kit (Aidlab, Beijing, China) following the manufacturer's instructions. An equal quantity of RNA from all three of the treatments and three independent biological replicates was blended for cDNA library construction to obtain the transcriptome data. The sequencing libraries were generated using NEBNext Ultra Directional RNA Library Prep Kit for Illumina (NEB, USA) following manufacturer's recommendations, and index codes were added to attribute sequences to each sample.

### Clustering and sequencing

The index-coded samples were clustered on a cBot Cluster Generation System using TruSeq PE Cluster Kit v3-cBot-HS (Illumina) according to the manufacturer's instructions. After the cluster generation, the library preparations were sequenced on an Illumina HiSeq 2000 platform, and paired-end reads were generated.

### Sequence read mapping, assembly and SSR detection

The raw data (raw reads) in fastq format were first processed through in-house Perl scripts. In this step, clean data (clean reads) were obtained by removing the reads containing adapters, reads containing ploy-N and low-quality reads from the raw data. At the same time, the Q20, Q30, GC-content and sequence duplication level of the clean data were calculated. All of the downstream analyses were based on high-quality clean data. The clean reads were assembled using Trinity software as described for de novo transcriptome assembly without a reference genome. The SSRs of the transcriptome were identified using MISA (http://pgrc.ipk-gatersleben.de/misa/misa.html).

### Gene expression quantification and differential expression analysis

The libraries were sequenced, and 50-bp single-end reads were generated. The clean data were mapped back onto the assembled transcriptome, and the read count for each gene was obtained from the mapping results. The gene expression levels were measured using the reads per kilobase per million reads (RPKM) method using the formula that was previously described by Mortazavi [47]. A differential expression analysis of three conditions was performed using the DESeq R package (1.10.1). Three independent biological replicates for each treatment were analyzed. The p values were adjusted using the Benjamini & Hochberg method. The corrected p value of 0.05 was set as the threshold for significantly differential expression.

### Functional annotation

The GO enrichment analysis of the DEGs was implemented by the GOseq R package-based Wallenius noncentral hypergeometric distribution [48], which can adjust for gene length bias in DEGs. The KEGG pathway enrichment analysis of the DEGs was performed using KOBAS [49].

### Validation of the DEGs by quantitative real-time PCR

For the quantitative RT-PCR of the mRNAs, 1 µg of total RNA was used to synthesize the cDNA using the PrimeScript RT reagent Kit (Takara, Japan). Real-time PCR was performed using SYBR premix Ex Taq (Takara, Japan) according to the manufacturer's instruction. The PCR amplification was performed under the following conditions: 95°C for 30 s, followed by 40 cycles of 95°C for 5 s and 60°C for 10 s. The products were verified by melting curve analysis. Quantification was achieved by normalizing the number of target transcripts copies to both *Actin* and *GAPDH* genes using the normalized expression method. Three independent biological replicates for each sample and three technical replicates for each biological replicate were analyzed. All of the primers that were used are listed in Table S6.

## Supporting Information

Table S1 **Summary of the Unigenes annotation.**
(DOCX)

Table S2 **Detailed frequencies of the EST-SSR repeat motifs.**
(DOCX)

Table S3 **KEGG pathway enrichment results by DEGs from mild stress versus control.**
(XLS)

Table S4 **KEGG pathway enrichment results by DEGs from severe stress versus control.**
(XLS)

Table S5 **KEGG pathway enrichment results by DEGs from severe stress versus mild stress.**
(XLS)

Table S6 **Primers for the qRT-PCR analysis.**
(DOC)
