# Supplementary material for: De Novo Assembly and Discovery of Genes That Are Involved in Drought Tolerance in Tibetan Sophora moorcroftiana
Source: PLoS One. 2015 Jan 5;10(1):e111054. doi: 10.1371/journal.pone.0111054 (PMC4283959; doi:10.1371/journal.pone.0111054)
Supplement: Table S1 — Summary of the Unigenes annotation. (DOCX) [file pone.0111054.s001.docx]

Table S1: Summary for the unigenes annotation

|  | Number of Unigenes | Percentage (%) |
| --- | --- | --- |
| Annotated in NR | 31117 | 47.12 |
| Annotated in NT | 20646 | 31.26 |
| Annotated in KO | 5377 | 8.14 |
| Annotated in SwissProt | 21493 | 32.55 |
| Annotated in PFAM | 20537 | 31.1 |
| Annotated in GO | 23310 | 35.3 |
| Annotated in KOG | 11801 | 17.87 |
| Annotated in all Databases | 2359 | 3.57 |
| Annotated in at least one Database | 34663 | 52.49 |
| Total Unigenes | 66026 | 100 |
