# Supplementary material for: De Novo Assembly and Discovery of Genes That Are Involved in Drought Tolerance in Tibetan Sophora moorcroftiana
Source: PLoS One. 2015 Jan 5;10(1):e111054. doi: 10.1371/journal.pone.0111054 (PMC4283959; doi:10.1371/journal.pone.0111054)
Supplement: Table S2 — Detailed frequencies of the EST-SSR repeat motifs. (DOCX) [file pone.0111054.s002.docx]

Table S2: Frequency of EST-SSR repeat motifs in *S. moorcroftiana*

| Repeat | Repeat numbers | | | | | | | Total (%) | |
| --- | --- | --- | --- | --- | --- | --- | --- | --- | --- |
| motif | 5 | 6 | 7 | 8 | 9 | 10 | >10 |  |  |
| AC/GT | 178 | 136 | 98 | 62 | 47 | 20 | 2 | 543 | 12.01% |
| AG/CT | 437 | 244 | 206 | 182 | 190 | 63 | 4 | 1326 | 29.34% |
| AT/AT | 145 | 69 | 49 | 22 | 8 | 9 | 2 | 304 | 6.73% |
| CG/CG | 2 | 4 | 1 | 1 | 1 | - | - | 9 | 0.20% |
| AAC/GTT | 184 | 97 | 34 | 1 | - | - | 1 | 317 | 7.01% |
| AAG/CTT | 307 | 170 | 87 | 4 | - | - | - | 568 | 12.57% |
| AAT/ATT | 139 | 57 | 34 | 3 | - | - | 1 | 234 | 5.18% |
| ACC/GGT | 172 | 64 | 24 | 4 | - | - | - | 264 | 5.84% |
| ACG/CGT | 16 | 12 | 4 | 1 | - | - | - | 33 | 0.73% |
| ACT/AGT | 20 | 10 | 5 | 3 | - | - | - | 38 | 0.84% |
| AGC/CTG | 103 | 35 | 16 | 5 | - | - | - | 159 | 3.52% |
| AGG/CCT | 124 | 35 | 16 | 4 | - | - | - | 179 | 3.96% |
| ATC/ATG | 213 | 76 | 38 | 5 | - | - | - | 332 | 7.35% |
| CCG/CGG | 57 | 9 | 6 | 3 | - | - | - | 75 | 1.66% |
| AAAC/GTTT | 6 | 1 | - | - | - | - | - | 7 | 0.15% |
| AAAG/CTTT | 18 | 3 | - | - | - | 1 | - | 22 | 0.49% |
| AAAT/ATTT | 37 | 3 | - | - | - | - | 1 | 41 | 0.91% |
| AACT/AGTT | - | 2 | - | - | - | - | - | 2 | 0.04% |
| AAGG/CCTT | 1 | 1 | - | - | - | - | - | 2 | 0.04% |
| AAGT/ACTT | - | 1 | - | - | - | - | - | 1 | 0.02% |
| AATC/ATTG | 1 | - | - | - | - | - | - | 1 | 0.02% |
| AATG/ATTC | 5 | - | - | - | - | - | - | 5 | 0.11% |
| AATT/AATT | 2 | - | - | - | - | - | - | 2 | 0.04% |
| ACAT/ATGT | 6 | 5 | - | - | - | 1 | - | 12 | 0.27% |
| ACGC/CGTG | - | 1 | - | - | - | - | - | 1 | 0.02% |
| ACTC/AGTG | 2 | 1 | - | - | - | - | - | 3 | 0.07% |
| AGAT/ATCT | 12 | 1 | - | - | - | - | - | 13 | 0.29% |
| AGCC/CTGG | 1 | - | - | - | - | - | - | 1 | 0.02% |
| AGGG/CCCT | 4 | - | - | - | - | - | - | 4 | 0.09% |
| ATCC/ATGG | 1 | - | - | - | - | - | - | 1 | 0.02% |
| AAACC/GGTTT | 2 | - | - | - | - | - | - | 2 | 0.04% |
| AAAGG/CCTTT | 1 | - | - | - | - | - | - | 1 | 0.02% |
| AACAC/GTGTT | 1 | - | - | - | - | - | - | 1 | 0.02% |
| AAGAG/CTCTT | 1 | - | - | - | - | - | - | 1 | 0.02% |
| AAGAT/ATCTT | 1 | - | - | - | - | - | - | 1 | 0.02% |
| AAGGG/CCCTT | 1 | - | - | - | - | - | - | 1 | 0.02% |
| AATCT/AGATT | 1 | - | - | - | - | - | - | 1 | 0.02% |
| AATGC/ATTGC | 1 | - | - | - | - | - | - | 1 | 0.02% |
| AATGG/ATTCC | 1 | - | - | - | - | - | - | 1 | 0.02% |
| AATGT/ACATT | 1 | - | - | - | - | - | - | 1 | 0.02% |
| AATTC/AATTG | 1 | - | - | - | - | - | - | 1 | 0.02% |
| ACACC/GGTGT | 1 | - | - | - | - | - | - | 1 | 0.02% |
| ACTCC/AGTGG | 1 | - | - | - | - | - | - | 1 | 0.02% |
| AGATG/ATCTC | 1 | - | - | - | - | - | - | 1 | 0.02% |
| AGCAT/ATGCT | 1 | - | - | - | - | - | - | 1 | 0.02% |
| ATATC/ATATG | 1 | - | - | - | - | - | - | 1 | 0.02% |
| AAAACC/GGTTTT | - | - | - | - | - | - | 1 | 1 | 0.02% |
| AACTGG/AGTTCC | - | 1 | - | - | - | - | - | 1 | 0.02% |
| ACAGCC/CTGTGG | - | - | - | - | 1 | - | - | 1 | 0.02% |
| ATCGCC/ATGGCG | - | - | 1 | - | - | - | - | 1 | 0.02% |
| Total | 2210 | 1038 | 619 | 300 | 247 | 94 | 12 | 4520 | - |
| （%） | 48.89% | 22.96% | 13.69% | 6.64% | 5.46% | 2.08% | 0.27% | - | - |
