# Supplementary material for: De Novo Assembly and Discovery of Genes That Are Involved in Drought Tolerance in Tibetan Sophora moorcroftiana
Source: PLoS One. 2015 Jan 5;10(1):e111054. doi: 10.1371/journal.pone.0111054 (PMC4283959; doi:10.1371/journal.pone.0111054)
Supplement: Table S6 — Primers for the qRT-PCR analysis. (DOC) [file pone.0111054.s006.doc]

**Table S6: Primers for qRT-PCR analysis**

| **Accession No.** | **Annotation** | **Primer set** | |
| --- | --- | --- | --- |
|  |  | **Forward primer (5’-3’)** | **Reverse primer (5’-3’)** |
| comp34365_c0 | DREB1 | caaaatactggcaaagtggaaa | acctttcatacaccccttcct |
| comp23267_c0 | DREB2 | ACAAGGGGATATGTCTGCTGATT | TCGGGTTTTGGATACTCGTTC |
| comp40161_c0 | DREB3 | GCCAGAAACGGTGTCGGT | GCTTGCATCAGCAAAAGTAAGAT |
| comp41730_c0 | DREB4 | ATGGCAGCAGCTATAGATATC | TCAAATAGCCTCCCAATCAAT |
| comp46334_c0 | AQP1 | ACCGTTTTGGCTGATGCTATT | CGTGGTCCCCGTCCTACTT |
| Comp43050_c0 | AQP2 | CACTGAAGCCGCAGCACA | GCCAATGACGGTCAAAACG |
| comp35177_c0 | AQP3 | CTTTGGTATCACTCTGGCTCTTG | CTCCTCCTGCACCCCTCA |
| Comp22192_c0 | CHI1 | TTTCATAAATACGCATAGTGGGG | CATTAGAAGCGGTTCTAGAAAGTGT |
| comp41215_c0 | CHI2 | CTGGCTCATCTATCCTCTTCACA | GCAGGGGAGACACCATCCT |
| Comp34535_c2 | PRX | TGAGAAGACCGAGAAAGCAGAC | TTATGACACCAAGGTCCAACG |
| Comp38763_c0 | POD | CCTCAGCAACCTTATCTCAACG | TCACATCCAGGTCCACCTTATT |
| Comp25556_c0 | ZFPK | TGGATGAGGGCCTTCTGTAAT | CAGGGATGAATGTGGAAAGTAAA |
| comp21317_c0 | ZFP | GGGCTCGCAAGTACAGGG | CGGGGCTAGGCTAAGGTG |
| Comp42823_c0 | CHS | TCCGAGATTACTGCTGTCACTTT | GGGATGCCGACACCAACT |
| Comp27454_c0 | MYB | ATTTCGTTCTTCGGCTTTCTC | TGAGCTGAAGTTTGATTTTGGAT |
| Comp24471_c0 | WRKY | GCTCCAGGGTTCATTCACAGA | CTACCGGGGTTTAGAGTCGG |
| comp48004_c2 | NAC | TCGCTCTGCAAGACTCATACAC | GCTTTCATGGCTAGACGAAGTT |
| Comp34868_c0 | ERF | CCTTGTCTGCTCATAAATCCACA | GTCACCAGTTTCAGTCCTTTGG |
| Comp23345_c0 | ABR | TGGCAAATCCTGATTACTAGACC | CGAAGTACAGACCATAAGGCAAC |
| Comp36883_c0 | SUT | ATAGCGGCAAGCAAAGGC | ACGAAAGGGGATATTGGGAG |
| Comp35373_c0 | CYP | GCCATTCTTGTTCCTTTGACTC | AGAAGAAACGAACCTCCCATTAG |
| Comp96748_c0 | FAR1 | ACGTCAAGCCAATAGAAGACAAA | GAGGTGCCGAACAAGATGC |
| Comp34161_c0 | GDPH | TAACAGGCAAAGTGAGAAAGGG | CCAATAGCAAGGAGGAGGCT |
| Comp32526_c0 | AIP | CCATAAGTCATAACTTGCTCATTCA | CCTGAGTGGACCATTCCTGTAACAT |
| comp34125_c0 | DHN | TGAACAGAACAAGGAAGGGATT | CACGGTGCTCACCTTTGTAGTC |
| comp37243_c0 | GAPDH | ATCAAGGAGGAGTCTGAGGGC | TGTCATACCATGCAACGAGCT |
| Previously identified | Actin | ttgttagggatgtgaaggaaaag | gaacctctggacatctgaaacg |
